# 1. A Case of Choking. 2. Inversion of Uterus and Vagina

**Published:** 1899-07

**Authors:** Walker S. Phillips

**Affiliations:** Reading, Pa.


### 1. A CASE OF CHOKING. 2. INVERSION OF UTERUS AND VAGINA.

By Dr. Walker S. Phillips, of Reading, Pa.

1. A Case of Choking. A few days before I was called a few miles away from the city, and found a fine milch cow laboring under symptoms of choking. Placed the balling-iron into her mouth, after giving several ounces of linseed oil; then introduced my hand into the back part of the mouth and throat, and found what I supposed to be part of an ear of corn. The animal was very much distressed, and I thought suffocation would take place. After several efforts to remove it, without avail, and thinking it had partly entered the trachea, I performed tracheotomy high up, introducing my finger, thinking I could remove the foreign body by pushing it forward, but could not reach to the body. The animal appeared relieved. I left her in the owner's care, with several small doses of oil to be given through the day. I heard nothing from the owner for several weeks, when I was called to the country, and had to pass through his barnyard, and, to my astonishment, there stood the cow among the herd as well as any of them.

2. Inversion of the Uterus and Vagina. I introduce three hip sutures, leaving the ends above and below, five or six inches long, after knotting them well together and drawing pretty tightly, then tying the above and lower ends together. Dieting the animal and giving chloroform and laudanum, three or four doses, which lessens the straining, leaving the sutures remain six or seven days. The sutures placed in the skin only, the parts are disfigured and lacerated in some cases, and make an unsightly appearance. I do not think there a better remedy when compelled to leave an animal six or eight miles away from you in the country, although it may not look scientific. I have used trappings of all sorts, and find animals sit down on their haunches and expel the parts.

